# Inference of temporally varying Bayesian Networks

**DOI:** 10.1093/bioinformatics/bts614

**Published:** 2012-10-16

**Authors:** Thomas Thorne, Michael P. H. Stumpf

**Affiliations:** Centre of Integrative Systems Biology and Bioinformatics, Division of Molecular Biosciences, Imperial College London, London SW7 2AZ, UK

## Abstract

**Motivation:** When analysing gene expression time series data**,** an often overlooked but crucial aspect of the model is that the regulatory network structure may change over time. Although some approaches have addressed this problem previously in the literature, many are not well suited to the sequential nature of the data.

**Results:** Here, we present a method that allows us to infer regulatory network structures that may vary between time points, using a set of hidden states that describe the network structure at a given time point. To model the distribution of the hidden states**,** we have applied the Hierarchical Dirichlet Process Hidden Markov Model, a non-parametric extension of the traditional Hidden Markov Model, which does not require us to fix the number of hidden states in advance. We apply our method to existing microarray expression data as well as demonstrating is efficacy on simulated test data.

**Contact:** thomas.thorne@imperial.ac.uk

## 1 INTRODUCTION

The analysis of gene expression data in the field of systems biology is a challenging problem for a number of reasons, not least because of the high dimensionality of the data and relative dearth of data points. A number of approaches have been taken to inferring regulatory interactions from such data, often using graphical models or sparse regression techniques (Lèbre, 2009; Opgen-Rhein and Strimmer, 2007; Schäfer and Strimmer, 2005).

These problems are further compounded by the nature of the biological systems under consideration, owing to the influence of unobserved actors that may alter the behaviour of the system. Often experiments are performed for long periods during which it is natural to expect the regulatory interactions at work to change. The time scales of regulatory responses to stimuli often differ from those of signalling and metabolic responses, and so it may be that responses to stimuli, around which experiments are often designed, take place in several phases each having different time scales.

Previous studies have attempted to address this problem by introducing changepoints in the time series, allowing the inferred network structure to differ between the resulting segments of the time series. For example in Lèbre *et al.* (2010), a changepoint model is applied in which Dynamic Bayesian Networks are inferred for each segment of the time series. However, such approaches may place strong prior assumptions on the number of changepoints that can be observed, and do not adjust for the complexity of the observed data automatically. Instead in Grzegorczyk *et al.* (2008), an allocation sampler is used in combination with Bayesian Networks to assign each observation to a group, but unlike changepoint models, this method treats the observations as being exchangeable, ignoring the fact that the data are sequential. The similar methodology in Ickstadt (2011) uses a more flexible non-parametric prior on group assignments, applied to the modelling of molecular interactions using Bayesian Networks, but suffers the same drawbacks in not recognizing the sequential nature of the data. A solution to the related, but different problem of inferring networks from multiple datasets that may vary in their underlying structure owing to changes in conditions, is presented in Penfold *et al.* (2012). By applying a hierarchical model, it is possible to model the interactions that may be shared for a number of different experimental conditions while also modelling the interactions specific to certain cases. However, this method treats the whole time series for a condition as a single static network, rather than allowing the network structure to change within a time series.

Here, we present a methodology that allows us to infer network structures that may change between observations in a non-parametric framework while modelling the sequential nature of the data. To that end, we have used the infinite hidden Markov model of Beal *et al.* (2002), also known as the hierarchical Dirichlet Process Hidden Markov Model (HDP-HMM) (Teh and Jordan, 2010), in particular the ‘Sticky’ extension of Fox *et al.* (2009), in conjunction with a Bayesian network model of the gene regulatory network structure. The HDP-HMM allows the number of different states of the network structure to adapt as necessary to explain the observed data, including a potentially infinite number of states, of course restricted in practice by the finite number of experimental observations. In the previous work of Rodriguez *et al.* (2010), it was demonstrated that the HDP-HMM outperforms a Dirichlet Process mixture for Gaussian graphical models on heterogeneous time series.

We apply our methodology to both simulated data and gene expression data for *Arabadopsis thaliana* and *Drosophila melanogaster*, demonstrating its effectiveness in detecting changes in network structure from time series data, and compare its performance and accuracy to existing methods. We also consider the biological implications of our results and present hypotheses as to their significance.

## 2 APPROACH

Given gene expression time series data over *m* genes at *n* time points, we denote the observations as the 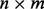 matrix 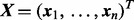, where 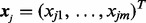, the column vector of expression levels for each of the *m* genes at time point *j*. We formulate our model as a HDP-HMM, a stochastic process, whereby a set of hidden states 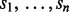 governs the parameters of some emission distribution *F* over a sequence of time points 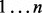.

Each observation 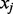 is then generated from a corresponding emission distribution 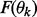, where 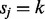. For our emission distributions, *F*, we use a Bayesian Network model over the *m* variables to represent the regulatory network structures corresponding to each hidden state.

## 3 METHODS

In the following, we will consider the problem of network inference in a Bayesian framework, aiming to draw samples from the distribution of the model parameters θ, given some observed data *X*, *P*(θ|*X*), known as the posterior distribution. By application of Bayes rule, it can be shown that for a given model
(1)
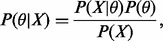

where the term *P*(*X*), commonly called the evidence, is constant with respect to the parameters θ, and so 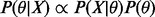. The prior distribution *P*(θ) over the parameters summarizes our knowledge of the model parameters before we have observed the data, and so it should be consistent with any data we could potentially observe.

### 3.1 The Dirichlet Process

Bayesian non-parametrics aims to ensure that the prior of a model remains appropriate for a wide range of data, allowing the complexity of an inferred model to adapt in light of the observed data. One particular Bayesian non-parametric formulation, known as the Dirichlet Process (an extension of the Dirichlet distribution as described below), has been used extensively as a prior in clustering and mixture modelling, as it is able to adapt the complexity of the model to best fit the number of components in the data, without resorting to schemes such as reversible jump Markov Chain Monte Carlo (MCMC) (Green, 1995), as used in Lèbre *et al.* (2010).

The Dirichlet Process is a non-parametric extension of the Dirichlet distribution (Gelman *et al.*, 2004), which can be constructed in a number of ways. Conventionally, the Dirichlet distribution is defined for *M* dimensional vectors *x* under the constraint that all 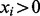 and 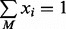, and takes parameters α*_i_*, for 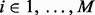:
(2)
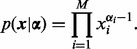



As the *x_i_* sums to one, they can be interpreted as specifying a discrete probability distribution over a set of outcomes 1, … , *M*. Using the Dirichlet distribution as the prior for a set of multinomial observations, α*_i_* can be interpreted as the number of a priori observations of outcome *i* (Gelman *et al.*, 2004). The Dirichlet Process can then be obtained as the limit of a symmetrical Dirichlet distribution with dimension *M* and concentration parameters 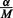 as *M* →*∞*.

One construction of the Dirichlet Process is the ‘stick breaking’ construction of Sethuraman (1994), whereby an infinite sequence of discrete probability *atoms* δ_θ_ are drawn from the underlying distribution, known as the base measure. These points are weighted by coefficients β*_i_*, that are defined as
(3)
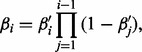

with
(4)
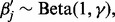

for some concentration parameter γ. The β*_i_* can thus be seen as lengths broken from a stick of unit length, β_1_ taking a length of 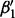, β_2_ taking a fraction 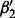 of the remaining stick (which has length) 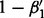 and so on. Larger values of γ will result in smaller values of 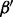 and thus many atoms 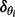 with similar weights β*_i_*. The distribution of the β*_i_* dependent on γ is referred to as 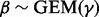.

Then for a Dirichlet Process with *concentration parameter* γ and *base measure H*, written *DP*(γ,*H*), and 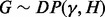, we have
(5)
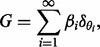

with 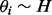 and 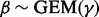.

As we will see with the application of the HDP to HMMs, the ability of Bayesian non-parametric methods to adaptively explain the complexity of the observed data makes them a valuable tool in the statistical analysis of data when we wish to make few a priori assumptions.

The HDP is constructed simply by taking a Dirichlet Process as the base measure of another Dirichlet Process. Then we have that
(6)
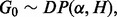

(7)
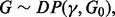



and using the stick breaking construction,
(8)
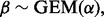

(9)
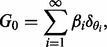

(10)
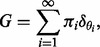

where 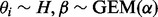 and 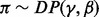. For a derivation of this form of the HDP, we refer the reader to Teh *et al.* (2006).

### 3.2 HDP-HMMs

To model a hidden state sequence that evolves over time, we apply the methodology first introduced in Beal *et al.* (2002), whereby a finite state Hidden Markov Model (HMM), consisting of a set of hidden states 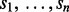 over some alphabet 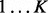, is extended so that *K* →*∞*. In a classical HMM (Bishop, 2006), the number of states *K* is typically specified in advance, and states follow a Markov process, whereby transitions are made between states with probability 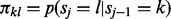 so that the next state in the sequence depends only on the previous state.

The HDP-HMM (Beal *et al.*, 2002; Teh *et al.*, 2006; Teh and Jordan, 2010) instead applies a *Dirichlet Process prior* to the transition probabilities 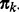 out of each of the states *k*, and uses a hierarchical structure to couple the distributions between the individual states to ensure a shared set of potential states into which transitions can be made across all of the 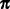. This allows for an unlimited number of potential states, of course limited in practice by the number of observed data points.

More formally, each hidden state *k* possesses a Dirichlet Process distributed *G_k_*, from which the next state is drawn, and a common (discrete) base measure *G*_0_ is shared between these Dirichlet Processes so that 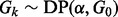. As a result, transitions are made into a discrete set of states shared across all of the *G_k_* and drawn from *G*_0_. The base measure *G*_0_ is in turn drawn from a Dirichlet Process, 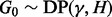, *H* being our prior over parameters for the emission distributions *F_k_*.

**Fig. 1.**
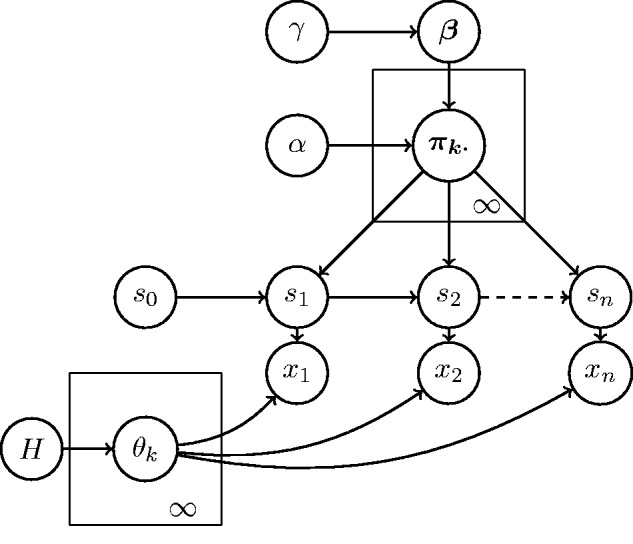
The HDP-HMM represented as a graphical model

Then using the stick-breaking construction of Sethuraman (1994) for *G*_0_ and drawing θ*_l_* independently from *H*, we have that 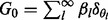, with 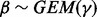, and so 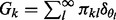 with 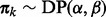. The resulting model is outlined in Figure 1. For a comprehensive introduction to the construction of the HDP-HMM, we refer the reader to the excellent and extensive description in Fox *et al.* (2009).

However, in a biological system, it is more realistic to assume that only a subset of the large variety of potential behaviours of the hidden state sequence is relevant, as behaviour such as rapid cycling between states at adjacent time points would a priori seem to be unlikely to be observed in most gene expression datasets. Thus, we choose to apply the Sticky HDP-HMM (Fox *et al.*, 2008, 2009), which introduces an extra parameter κ that biases the prior probability of transitions between states towards remaining in the current state rather than transitioning to a differing one. Adding such a prior assumption simply states that we expect the state of the system to remain the same between successive time points; this is both parsimonious and would seem to be justified in the case of gene expression datasets, where we might only expect to observe a small number of transitions to differing states across the time series.

This modification to the HDP-HMM gives us a model generating observed data points *x_j_* as (Fox *et al.*, 2009)
(11)
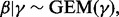

(12)
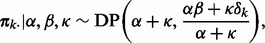

(13)
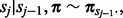

(14)
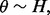

(15)
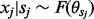



### 3.3 Gibbs sampling for the Sticky HDP-HMM

To sample from the hidden state sequence, we have used a Gibbs sampling procedure (Robert and Casella, 2005) based on the conditional probabilities for the hidden state *s_i_*_,_ given the remaining hidden states 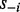 as described in Fox *et al.* (2008), updating each hidden state individually in a sweep over the *n* states,

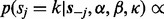


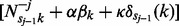


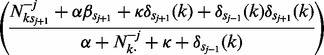

(16)
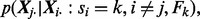

where 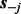 denotes the state sequence 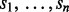 excluding *s_j_*, 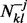 indicates the number of observed transitions from state *k* to state *l* within the hidden state sequence 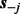, and 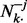 the total number of transitions from state *k* within 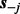.

Briefly, to update the hidden state sequence *s*, iterating over each *j*, 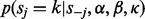 is calculated for all *k*, and a weighted sample taken from these to decide the updated value of *s_j_*. The full process is described in Algorithm 1. We use standard vague prior parameters for α and β (Dunson, 2010), and set κ so as to prefer sequences of identical consecutive states. It is possible in principle to further extend the method by adding priors on the hyperparameters α, β and κ, but in most cases, the HDP-HMM already exhibits the required flexibility without this.

### 3.4 Bayesian Network emission distributions

To model the regulatory network structure corresponding to the hidden states of the HDP-HMM, we have applied a Bayesian Network methodology to capture the relationships between the genes represented in our data. Thus, each hidden state has a unique Bayesian Network describing the interactions occurring between the genes at the time points corresponding to a particular state. Bayesian Networks are probabilistic models, whereby a directed graph defines the conditional independence relationships between a set of random variables (Koski and Noble, 2009). For the model to remain consistent, the graph structure 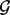, with nodes 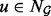 representing random variables and directed edges 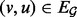 representing conditional probability relationships between them, must be acyclic.

For a given Bayesian network structure, 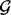, and model parameters, 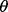, the joint distribution 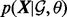 factorizes as a product of local distributions for each node,
(17)
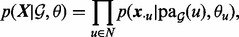

where for each observation, the value *x_iu_* of a node *u* is dependent on the values of its set of parent nodes 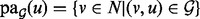 and some parameters θ*_u_*. Here, we have used a Gaussian Bayesian Network (BGe) model (Geiger and Heckerman, 2002) that allows the variables to take continuous values and defines the local distributions for each observation 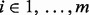 of a gene *u* as
(18)
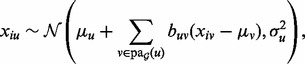

with parameters 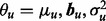. With a Wishart distribution, the conjugate before the multivariate Normal distribution, this simplifies the form of the resulting equations, and we can calculate the local marginal likelihoods 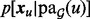 as described in Geiger and Heckerman (2002) and from these derive the joint probability 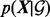.

Unfortunately, owing to the restriction of the network structure to that of a directed acyclic graph (DAG), it is difficult to explore the space of possible network structures. Several MCMC schemes have been proposed, including those of Grzegorczyk and Husmeier (2008) and Madigan *et al.* (1995), but performing random walks over DAG network structures faces the problem that proposing moves that maintain the DAG structure can be complex and time consuming, and mixing of the Markov chain can be slow. However, as noted in Friedman and Koller (2003), a DAG structure 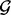 corresponds to a partial ordering on the nodes and so induces a (non-unique) total ordering, and allows us to perform a random walk over total orderings of the nodes. This Markov chain efficiently explores the space of possible graph structures, improving the mixing properties of the chain.

Although this introduces a bias in the prior distribution over graph structures (Grzegorczyk and Husmeier, 2008), it greatly simplifies the computational complexity of the MCMC procedure, and such a bias may be justified by arguments of parsimony, as graphs consistent with more orderings are more likely to be sampled. Furthermore, the uniform prior on DAG structures is not uniform over Markov equivalent graphs, and so also introduces a different kind of bias in the results. Finally, a trivial modification of the algorithm of Friedman and Koller (2003) allows for a correction of the bias (Ellis and Wong, 2008). Thus, in our methodology, we apply the MCMC sampler of Friedman and Koller (2003) to infer Bayesian Network structures for each hidden state of the HDP-HMM by sampling over total orderings of the nodes 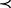, given the data points corresponding to the state in question. It is easy to calculate the likelihood of an ordering 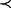 using the formula given in Friedman and Koller (2003)
(19)
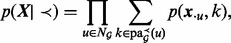

where 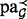 denotes the set of possible parent sets over the nodes of 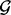 consistent with the ordering 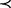. Then we can use a Metropolis Hastings sampler to sample from the posterior of orderings 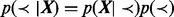 (Ellis and Wong, 2008), by beginning with an initial ordering and proposing and accepting new orderings 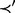, distributed as 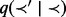 with probability according to the Metropolis Hastings acceptance probability
(20)
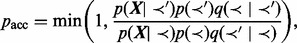

over a number of iterations. We choose to propose changes by swapping nodes in the ordering rather than more complex schemes such as ‘deck cutting’, as these were found to have little impact on performance in previous studies (Ellis and Wong, 2008; Friedman and Koller, 2003). Proposals 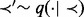 are thus drawn by selecting two nodes within the ordering uniformly at random and exchanging their positions to produce a new ordering. In the absence of a compelling alternative, we take the prior over orderings 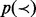 as the uniform distribution.

Then for our emission distribution for a given state *k*, we apply a Bayesian Network ordering 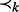 generating observed data points 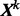 distributed as 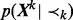 where by 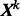 we denote the subset of *X_ij_*, including only rows *i* corresponding to states *s_i_* = *k*.

The full method is outlined in Algorithm 1 and combines Gibbs updates of the hidden state sequence with Metropolis Hastings updates of the node orderings of the Bayesian Networks for each state at every iteration. To sample hidden state sequences and orderings from the posterior distribution, the algorithm is first run for a number of burn-in iterations, after which samples are collected. As a single iteration of our algorithm combines a full Gibbs update sweep along with an update of the Bayesian Network orderings over a number of Metropolis Hastings steps, in practice a comparatively small number of iterations of the algorithm are required to reach the posterior. To reduce the computational complexity of the Bayesian Network inference, we restrict the number of potential parents of a gene to be ≤2. Even in such a case, we still face a large number of possible parent sets, of size 
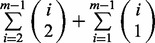
, and so in the analyses presented below, we restrict our dataset to a subset of genes of special interest, as is commonly the case in gene expression data analysis.

Given that the parent set for a given group of genes will be of size 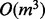, the computational complexity of performing Gibbs sampling over each of the data points will be 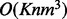, where *K* is the number of hidden states.

Finally, once we have inferred the hidden state sequence and generated a posterior sample of orderings corresponding to each state, we can then easily sample DAG structures from the posterior by first sampling an order from the posterior of a given state, and then sampling from the graphs consistent with this ordering, weighting the choice of parents by the local scores, and optionally attempting to account for the bias in the prior as described in Ellis and Wong (2008).


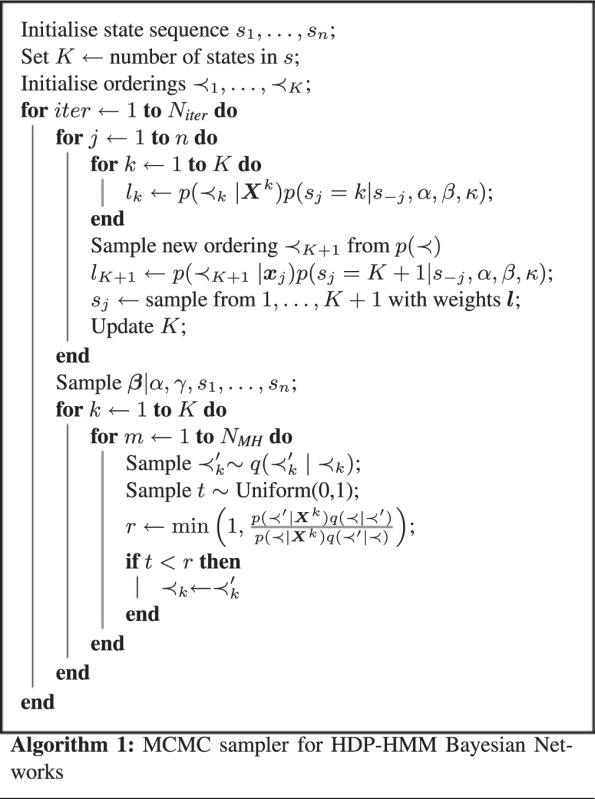


## 4 RESULTS

### 4.1 Example—simulated data

To evaluate the efficacy of our method, we generated simulated data from three different Bayesian network structures and interleaved the data points into a single time series. Applying our methodology to this data, we then attempted to recover the hidden state sequence.

Three different Bayesian Networks of 10 nodes each having random structures and parameters were used, with the restriction that each node had at most two parents. Such a restriction is realistic for real world biological networks and reduces the computational complexity of the Bayesian network inference, as the number of potential sets of parents of each node is greatly reduced by constraining the search. A total of 100 data points were used, consisting of a sequence of 25 generated by the first network, 25 by a second network structure, another 25 from a third network structure and finally a further 25 data points generated by the second network structure.

The Gibbs sampling MCMC scheme outlined in Algorithm 1 was applied over 500 iterations after a 1000 iteration burn in, with 100 MCMC iterations of the Bayesian network order sampler run on each network structure between each Gibbs sweep. We performed a comparison of the true hidden state sequence with the state sequences for the 500 samples from the Gibbs sampler, and found that our method perfectly recreates the original hidden state sequence, correctly identifying that the network structure is the same between two separate segments of the time series.

To assess the accuracy of our method, we compared its performance to the Auto Regressive TIme VArying (ARTIVA) method of Lèbre *et al.* (2010). Although ARTIVA was able to infer change points at the appropriate time points for one of the genes, all of the remaining genes had no predicted changepoints, despite the fact that their interactions change during the time series.

### 4.2 *Drosophila melanogaster* midgut development gene expression data

Applying our method to real world gene expression data, we took the publicly available gene expression dataset of Li and White (2003), as stored in the Gene Expression Omnibus database (Edgar *et al.*, 2002). This dataset gives tissue specific expression levels for genes in *D. melanogaster* midgut at time points before and after puparium formation, taken at 11 time points. A subset of genes to analyse was chosen by selecting genes having the highest variance across the time series, using the genefilter R package in Bioconductor www.bioconductor.org (Gentleman *et al.*, 2007; R Development Core Team, 2011). This resulted in a dataset of 23 genes at 11 time points. This allows us to apply our approach without having to consider the additional issues arising from the ‘large- *p*-small- *n*’ problem.

**Fig. 2.**
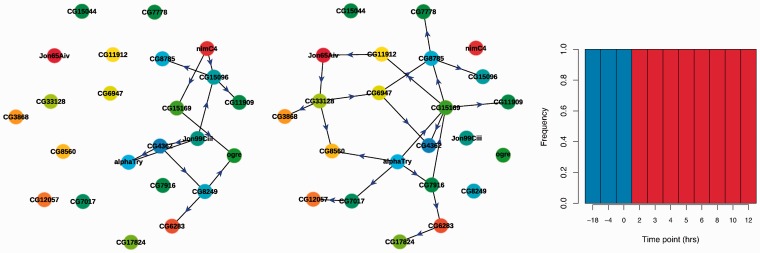
(Left) Inferred network structure corresponding to the first hidden state. (Middle) Inferred network structure corresponding to the second hidden state. (Right) Posterior distribution of states at each time point inferred by our method applied to the *D. melanogaster* midgut development expression data (Li and White, 2003). States are represented by colours, and frequencies of their appearance for each time point in the posterior samples are plotted. The first state is coloured blue, the second red

The results shown in Figure 2 identify two regions of the time series having different network structures, with a change occurring after the 0 hour time point at which puparium formation occurs. This suggests that a different structure of regulatory interactions is at work during the midgut development after the puparium formation begins. The networks inferred for each of the different states are also shown in Figure 2, illustrating a clear change between differing network structures. A main objective of this type of approach is to distill new mechanistic hypotheses from such data, and the temporally resolved and varying network structures do, indeed, deliver candidates for further analysis.

Looking at the inferred network structures, e.g. we see a number of genes whose interaction patterns change over the course of the time series. Perhaps most interesting amongst these are the genes Jonah 65Aiv (Jon65Aiv) and Jonah 99Ciii (Jon99Ciii), which are known to be expressed in the *D. melanogaster* midgut during development (Akam and Carlson, 1985), but whose function is not fully understood. It appears that Jonah 99Ciii is involved in development before puparium formation, whereas Jonah 65Aiv develops several interactions after puparium formation. The gene alphaTry seems to be involved in development before and after puparium formation, whereas nimrod C4 (nimC4) seems to interact only before puparium formation. In addition to this, a number of relatively unknown genes appear to have differing regulatory interactions between the time points. Given only gene expression data, it is not feasible, however, to identify potential mechanisms of the changes taking place, as many different factors may affect the presence or absence of regulatory interactions. The inferred network structure before puparium formation is based on a small number of time points, and so may not be entirely robust. However, such cases are bound to arise when considering time varying networks without a priori knowledge of the time varying structure, and should be treated as indications that further experimental work is needed if closer investigation of the network structure is required.

### 4.3 Transcriptome of starch metabolism during *A. thaliana* diurnal cycle

We have also analysed the gene expression dataset of Smith *et al.* (2004), as included in the GeneNet (Schafer and Opgen-Rhein, 2006) R package (R Development Core Team, 2011). The dataset consists of expression levels for 800 genes encoding enzymes involved in starch synthesis and in conversion of starch to maltose and Glc, at 11 time points for 12 h, transitioning from dark to light. The first 5 time points were collected during a dark period after which a switch to a light period was made, with time points spaced so that expression is measured at 0, 1, 2, 4 and 8 h after the switch to the dark period, and the same intervals after the switch to the light period (Smith *et al.*, 2004), as well as a final 24-h time point at the switch back to the dark period. A reduced subset of the 800 genes in the dataset was selected using the genefilter R package, as described previously, giving a subset of 40 genes that were analysed using our method.

**Fig. 3.**
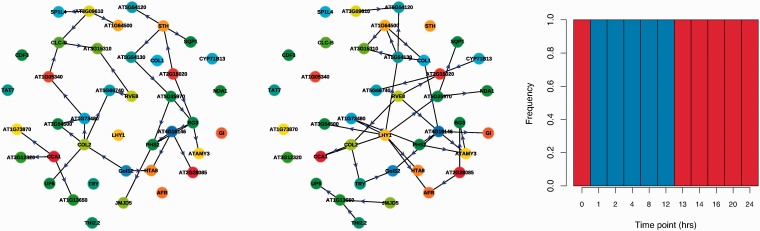
(Left) Inferred network structure corresponding to the first hidden state. (Middle) Inferred network structure corresponding to the second hidden state. (Right) Posterior distribution of states at each time point inferred by our method applied to the *A. thaliana* diurnal cycle expression data (Smith *et al.*, 2004). States are represented by colours, and frequencies of their appearance for each time point in the posterior samples are plotted. The first state is coloured blue, the second red

In Figure 3, we show the results generated by our method, clearly indicating two distinct phases within the time series. It appears that one phase is detected from 1 to 12 h, with a second phase inferred between 13 and 24 h that is also represented at the initial time point. This is consistent with the design of the experiment, as a change in expression would perhaps not be expected to be observed immediately at the point at which the switch between light and dark takes place, but rather later at a subsequent time point, as is observed here. As the 24-h time point was taken under the same conditions as the initial time point, one would expect these two time points to be grouped together using our method. The networks inferred for the two different phases, shown in Figure 3, again demonstrate a clear change in the network structure, with the two networks having distinct topologies.

Several of the genes, e.g. COL2 and CCA1, appear to interact both during the light and dark phases, and both are known to be involved in circadian regulation (Alabadí *et al.*, 2001; Ledger *et al.*, 2001). A gene showing a clear differentiation in its interactions between the dark and light phases is LHY1, with no interaction inferred during the dark phase, followed by a proliferation of interactions in the light phase. It is known that LHY1 is expressed at peak levels at dawn (Schaffer *et al.*, 1998) and involved in flowering, and mutants cause late flowering (Coupland *et al.*, 1998). AFR appears to be regulated by LHY1 during the light phase, and AFR is known to be involved in far-red light signalling (The Arabidopsis Information Resource (TAIR), www.arabidopsis.org).

## 5 DISCUSSION

From our simulated data, it appears that the HDP-HMM Bayesian Network sampler we have constructed accurately infers the hidden state sequences governing Bayesian Networks that capture how the regulatory organization of a biological system, here observed at the level of mRNA data, changes with time. By delivering time-resolved predictions of regulatory interactions, our method generates biological hypotheses that can be tested more robustly through the use of e.g. conditional knock-downs and RNAi. Further to this, network structures that are adopted for a small number of samples can identify segments of the time series, focussing on which would improve the modelling of the system, thus suggesting experiments that will deliver increased understanding of the biological system being examined. The accuracy of our method on test data lends hope that it will perform well on real world datasets, and the existence of more sophisticated and demonstrably more efficient samplers indicates that there is room for even further improvement and computational efficiency. For example, the beam sampler of Van Gael *et al.* (2008) and the Hierarchical Chinese Restaurant Process formalism of Makino *et al.* (2011) show improved mixing and perform better than standard Gibbs samplers, especially on time series, such as those we examine here where neighbouring states are likely to be correlated.

We would like to emphasize that it is essential to consider the fluid nature of regulatory network structures when inferring networks from datasets where such change is likely. Performing an analysis on data using a model with a fixed network structure, when it is known or believed that the network structure will change (this possibility should really never be discarded), is inherently incorrect, and thus will introduce unnecessary bias into the results. Although it may be possible to infer correct results from an incorrect model, it would not seem wise to rely on such approaches when alternatives exist.

Our methodology crucially accounts for the sequential nature of the data, something that has previously been ignored (Ickstadt, 2011; Grzegorczyk *et al.*, 2008), but we feel is crucial to the modelling of gene expression time series datasets. Furthermore, our methodology has an advantage over changepoint models that data may be shared between distinct segments of the time series sharing the same hidden state when inferring the network structure—something that is explicitly represented in our model, but generally omitted in changepoint models such as ARTIVA. This is especially important in gene expression data analysis where time points are a scarce and valuable resource.

Although our method is computationally expensive, this comes purely as a result of the Bayesian Network inference rather than the HDP prior. The HDP-HMM requires computation of the likelihood of each state for each timepoint, given the remaining data, but this requirement is common to all similar methods. Thus, our method is comparable in cost with other Bayesian non-parametric methods operating on Bayesian Networks (Ickstadt, 2011; Grzegorczyk *et al.*, 2008) and scales similarly. In many circumstances, the performance will be more robust if the question is sufficiently well formed, as whole-genome level analyses tend to be plagued by a number of statistical problems (Lèbre, 2009; Opgen-Rhein and Strimmer, 2007; Schäfer and Strimmer, 2005) that can be circumvented by more focussed analyses. In principle, however, whole-genome analysis is possible in the HDP-HMM framework.

The versatility of the HDP-HMM means that our methodology is applicable not only to time series data where the underlying process is divided into distinct contiguous segments, as would be expected in gene regulatory networks, but also to processes describable by a Markov process, e.g. rapidly changing between a sequence of hidden states with some underlying transition mechanism. Thus, it may be of use for other problems of network inference in systems biology outside of the area of sequential gene expression time series data, or in other fields where networks that change with time are encountered. Moreover, proteomic and other data can be included in the inferential framework (whence some of the hidden states, for example, now become part of the observed data too).

Finally, although other methods may require manual specification of an appropriate prior distribution on the number of possible states, taking a non-parametric approach allows our prior distribution to naturally expand to explain the observed data as the size and complexity of the data grows. Bayesian non-parametric methods demonstrably outperform regular priors in a variety of applications, and we have shown here their potential in modelling hidden variables in theoretical systems biology.

*Funding: *T.T. and M.P.H.S. gratefully acknowledge support from the BBSRC (BB/F005210/2). M.P.H.S. is a Royal Society Wolfson Research Merit Award holder.

*Conflict of Interest*: none declared.
